# Multifunctional rhodamine B appended ROMP derived fluorescent probe detects Al^3+^ and selectively labels lysosomes in live cells

**DOI:** 10.1038/s41598-020-76525-0

**Published:** 2020-11-11

**Authors:** Upendar Reddy Gandra, Raphael Courjaret, Khaled Machaca, Mohammed Al-Hashimi, Hassan S. Bazzi

**Affiliations:** 1 Department of Chemistry, Texas A&M University at Qatar, P.O.Box 23874, Doha, Qatar; 2 Department of Physiology and Biophysics, Weill Cornell Medicine Qatar, P.O. Box 24144, Doha, Qatar; 3 Department of Materials Science & Engineering, Texas A&M University, 209 Reed McDonald Building, College Station, TX 77843-3003 USA

**Keywords:** Cell biology, Chemistry

## Abstract

There a few reports of rhodamine-based fluorescent sensors for selective detection of only Al^3+^, due to the challenge of identifying a suitable ligand for binding Al^3+^ ion. The use of fluorophore moieties appended to a polymer backbone for sensing applications is far from mature. Here, we report a new fluorescent probe/monomer** 4** and its ROMP derived polymer **P** for specific detection of Al^3+^ ions. Both monomer **4** and its polymer **P** exhibit high selectivity toward only Al^3+^ with no interference from other metal ions, having a limit detection of 0.5 and 2.1 µM, respectively. The reversible recognition of monomer **4** and **P** for Al^3+^ was also proved in presence of Na_2_EDTA by both UV–Vis and fluorometric titration. The experimental data indicates the behavior of **4** and **P** toward Al^3+^ is pH independent in medium conditions. In addition, the switch-on luminescence response of **4** at acidic pH (0 < 5.0), allowed us to specifically stain lysosomes (pH ~ 4.5–5.0) in live cells.

## Introduction

The development of highly sensitive and selective fluorescent chemosensors over recent years has attracted great interest^1–7^. One such element of interest is aluminium being the third most abundant element worldwide, and being significantly utilised in various applications such as in the healthcare, manufacturing industries, food additives, kitchen utensils and packaging. Furthermore, elevated levels of Al^3+^ ions in the human body can result in serious health problems^7^. Given the extensive use of Al^3+^ these health effects need to be considered. Some causes of aluminium toxicity have been linked to aluminosis, dialysis encephalopathy, Alzheimer’s, Parkinson’s and breast cancer^6^. In addition, aluminum is found naturally in the environment and drinking water. The tolerable weekly intake of Al^3+^ set by the European food safety authority (EFSA) is 1 mg Al/kg body weight in a 60 kg adult and according to the World Health Organization (WHO) the concentration of Al^3+^ ions in drinking water should be lower than 7.41 μM^7^.

In this respect, and due to the potential impact of Al^3+^ on human health and the environment, it is of considerable importance to develop new fluorescent sensor probes. To date, most of the reported Al^3+^ probes developed are based on small organic molecules^8–25^, carbon dots^26–28^, MOFs^7,29^, and transition metal complexes^30,31^. However, many of such systems suffer from achieving specificity either due to the interference of other transition metal ions^4,9,32,33^ or weak coordination and strong hydration ability (enthalpies of hydration is − 4680 kJ/mol) character. Thus, the issue of specific, efficient recognition of Al^3+^ with colorimetric and fluorometric response is challenging one to attain semi quantification and higher sensitivity. In this contest, chemosensors appended to a polymeric material is an attractive way to engineer new probes, preserving their activity for longer times and promoting polymerization^34,35^. In addition, having a polymer backbone has several advantages: firstly, there would be a high signal amplification because of the increase in the number of receptor moieties attached to a single site. Secondly, polymers can be easily fabricated into several applications by incorporating several different fluorophores and recognition units into the polymer backbone.

Herein, we report the synthesis of probe **4** by reacting the highly tethered ring-strained dione^36^ with rhodamine B. Subsequently, ring opening metathesis polymerization (ROMP) as a simple methodology was utilized for the synthesis of polymer **P**. ROMP has many advantages over other polymerization technics since it is chemically robust and has excellent functional group tolerance^37–40^. To date, very few reports was reported by using norbornene appended fluorophore based polymers via ROMP strategy for sensing applications^41–46^. Both probe **4** and polymer **P** showed high selectivity and exhibited a large spectral response towards binding with Al^3+^. In addition, the switch-on luminescence response of probe **4** at acidic pH (0 < 5.0) allowed us to selectively label lysosomes in live cells. There are few disadvantages associated with the current reported lysosomal pH probes; including short excitation wavelengths, which considerably restricts the use of the probes in bio-imaging^47,48^ complicated synthetic routes, lack of specificity and poor photo stability^49,50^.

In this regard, we utilized rhodamine derivatives having amine group of low ionic strength with the ability to stain lysosomes having a high excitation wavelengths^49,51,52^. This can be used as a marker that is lysosome-specific, an alternative low-cost strategy in comparison to utilizing expensive commercial dyes for specific staining of lysosomes.

## Experimental

### Materials and instrumentation^36^

Rhodamine B, ethylene diamine, *exo*-3,6-Epoxy-1,2,3,6-tetrahydrophthalic anhydride, triethyl amine and Hoveyda-Grubbs 2nd generation catalyst (HG2). All metal nitrate salts such as NaNO_3_, KNO_3_, Mg(NO_3_)_2_, Al(NO_3_)_3_, Cu(NO_3_)_2_, Zn(NO_3_)_2_, Co(NO_3_)_2_, Ni(NO_3_)_2_, Zr(NO_3_)_3_, Ce(NO_3_)_3_, La(NO_3_)_3_, Cd(NO_3_)_2_, Hg(NO_3_)_2_, Pb(NO_3_)_2_ and LiNO_3_ were purchased from Sigma-Aldrich. Solvents were purified by standard techniques prior to use for all synthesis. ^1^H NMR and ^13^C NMR spectra were recorded on AV 400 MHz Bruker or AV 600 MHz Bruker NMR spectrometer using CDCl_3_ and CD_3_CN as the solvent at 298 K. Tetra methyl silane (TMS) as an internal standard for ^1^H NMR. GPC analysis were carried out using a Viscotek GPC Max VE 2001 instrument with Viscotek TDA 302 tripe array detector Viscotek Org Guard column. UV–Vis spectra were recorded on a Perkin Elmer Lambda 950 UV–Vis spectrometer, using quartz cells of 10 mm path length at 273 K. Fluorescence emission spectra were recorded on Cary eclipse fluorescence spectrophotometer, using quartz cells of 10 mm path length at 273 K. IR spectra on Perkin Elmer FT-IR spectrometer, DSC spectra on Perkin Elmer Jade DSC and TGA on Perkin Elmer Pyris 6 were recorded.

### General experimental methods for UV–vis and fluorescence studies^53,54^

20 × 10^–3^ M solution of the nitrate salts of the respective ion (Na^+^, K^+^, Fe^3+^, Na^+^, Mg^2+^, Ni^2+^, Co^2+^, Cu^2+^, Cd^2+^, Pb^2+^, Zn^2+^, Al^3+^, Ce^3+^, La^3+^ and Hg^2+^) were prepared in pure aqueous medium and the same solution was used for all the studies after appropriate dilution. A stock solution of the monomer **4** and polymer **P** was prepared in dimethylsulphoxide (DMSO) medium and 10 μL or 5 μL of this stock solution was added to 2.98 mL of HEPES aqueous buffer: acetonitrile (1:1) medium having solution pH 7.2 to make the effective ligand concentration of 10 µM or 5 µM. For all luminescence measurements, λ_Ext_ = 525 nm with an emission slit width of 2.5 nm.

### Cell lines

All cell lines were obtained from ATCC and are grown in high glucose DMEM (4.5 g L^−1^) supplemented with 10% FCS and penicillin (100 U mL^−1^) streptomycin (100 μg mL^−1^). The cells were plated 24 h before experiments at a density of 2000 cells mm^−2^ on glass bottom dishes coated with Poly-d-Lysine (Mattek).

### Staining

The culture media was replaced by a saline of the following composition (in mM): 145 NaCl, 5 KCl, 2 CaCl_2_, 1 MgCl_2_, 10 Glucose, 10 HEPES. The cells were incubated at room temperature with 25 μM of **4** diluted in the saline for 60 min and then washed three times prior to imaging. The nuclear stain Hoechst 33342 (10 μg mL^−1^) was added to the staining solution. In another set of experiments, the lysosomal compartment was stained using 0.5 μM of Lysotracker Blue DND-22 (Thermofisher) and the cells simultaneously loaded with 5 μM of **4** for 60 min. For cell imaging experiments, the probes were diluted in pure DMSO and further diluted to the final working concentration. Stock solutions were prepared so that the maximum final DMSO concentration was 0.5%.

### Imaging

The images were acquired using a confocal microscope (Zeiss LSM 880) fitted with a 40×/1.30 objective controlled by the Zen black software (ver. 2.3, Zeiss). The **4** was imaged using the following parameters: excitation λ_ext_ = 561 nm and detection λ_em_ = 566/685 nm, and for Hoechst λ_ext_ = 405 nm and λ_em_ = 410–542 nm, the pinhole was set to 1 Airy unit. The scattered light from the 405 nm excitation was collected using a transmission photomultiplier tube (T-PMT) to generate a bright field image of the cells. For the co-localization experiments in the lysosome the Airy Scan detector was used with the same laser lines and automatic post-processing of the image using the Zen software.

### Cell viability assay

The potential effect of the probes on cell viability was evaluated using a Thiazolyl Blue Tetrazolium Blue (MTT) assay. Hela cells seeded at a density of 7000 cells per well were treated for 1 h with various doses of the probes or the corresponding vehicle concentration (DMSO, max concentration 0.5%). The cells were then loaded with 0.25 mg mL^−1^ of MTT for 2 h at 37 ºC, the media was then removed, and the cells freeze-dried at − 80 ºC. The final product was resuspended in DMSO before reading the absorbance on a multiplate reader (Clariostar, BMG LabTech). The experiment was conducted once on 4 technical replicates.

## Synthetic procedures

### Procedure of synthesis of aminoethyl rhodamine B (2)^55^

Rhodamine B (1.0 g, 2.26 mmol) was dissolved in 30 mL of ethanol. It was then heated to 70 °C with constant starring. Then excess ethylene diamine (2.5 mL) was added to the reaction medium. It was then allowed to reflux at 75 °C for 14 h. After cooling, the reaction mixture solvent was removed in vacuum under reduced pressure. Subsequently water (20 mL) was added to the reaction mixture and the organic phase was extracted using DCM (3 × 20 mL), dried over MgSO_4_ and the pure product **2** was isolated as a light red solid in 94% yield. ^1^H NMR (400 MHz, CDCl_3_) *δ* 7.99–7.82 (1H, m), 7.44 (2H, dd, *J* = 5.6 Hz, 3.1 Hz), 7.09 (1H, d, *J* = 3.0 Hz), 6.43 (2H, d, *J* = 8.8 Hz), 6.38 (2H, d, *J* = 2.5 Hz), 6.27 (2H, dd, *J* = 8.9 Hz, 2.5 Hz), 3.33 (8 H, q, *J* = 7.1 Hz), 3.19 (2H, t, *J* = 6.6 Hz), 2.40 (2H, t, *J* = 6.6 Hz), 1.16 (12H, t, *J* = 7.0 Hz). ^13^C NMR [151 MHz, CDCl_3,_ 298 K] *δ* 168.58, 153.45, 153.25, 148.77, 132.38, 131.22, 128.67, 128.02, 123.80, 122.72, 108.09, 105.62, 97.65, 77.29, 76.87, 64.88, 44.32, 43.86, 40.80 and 12.57. IR (ATR) *ν* = 1680 and 1616 cm^−1^.

### 2-(6-((2-(3′,6′-bis(diethylamino)-3-oxospiro[isoindoline-1,9′-xanthen]-2-yl)ethyl)amino)hexyl)-3a,4,7,7a-tetrahydro-1H-4,7-epoxyisoindole-1,3(2H)-dione (**4**)^55^

Amino ethyl rhodamine B (600 mg, 1.23 mmol) was dissolved in 10 mL dry THF. To this Et_3_N (170 µL) was added and the resulting solution was stirred for 20 min under N_2_ atmosphere. Then 2-(6-bromohexyl)-3a,4,7,7a-tetrahydro-1H-4,7-epoxyisoindole-1,3(2H)-dione (403 mg, 1.23 mmol) was added and the resulting reaction mixture and refluxed for 12 h. TLC monitored the progress of the reaction. Upon completion of the reaction, reaction mixture was allowed to attain the room temperature. Solvent was removed in vacuum under reduced pressure. The crude reaction mixture washed with water (15 mL), and then extracted with DCM (3 × 15 mL). The organic solvent was concentrated; column chromatography was performed using silica gel (chloroform) to afford **4** in pure form (500 mg, 54%). ^1^H NMR [400 MHz, CDCl_3,_ 298 K] *δ* 7.89 (1H, dd, *J* = 5.8 Hz, 2.6 Hz), 7.49–7.40 (2 H, m), 7.08 (1H, dd, *J* = 5.8 Hz, 2.6 Hz), 6.50 (2 H, s), 6.39 (4 H, dd, *J* = 17.9 Hz, 5.6 Hz), 6.27 (2 H, dd, *J* = 8.8 Hz, 2.5 Hz), 5.25 (2 H, s), 3.45 (2 H, dd, *J* = 14.1 Hz, 6.8 Hz), 3.41–3.24 (12 H, m), 2.58–2.41 (3 H, m), 2.22 (1H, s), 1.62–1.48 (2 H, m), 1.41 (2 H, s), 1.23–1.10 (17 H m); ^13^C NMR [151 MHz, CDCl_3_] *δ* 176.3, 170.7, 153.62, 149.18, 136.54, 133.52, 129.64, 128.57, 124.10, 123.08, 108.48, 103.18, 97.88, 80.91, 77.27, 76.85, 67.07, 48.89, 47.66, 44.40, 38.87, 31.58, 29.69, 27.29, 26.33, 25.91, 22.65, 14.12 and 12.59; LCMS calcd.for C_44_H_53_N_5_O_5_: 731.4, found: 732.6 [**4** + H^+^]; IR (ATR) *ν* = 1692, 1676, 1630 and 1610 cm^−1^.

### Polymerization of 4 in presence of [HG2]^36^

In a glove box, **4** (100 mg, 0.137 mmol) was dissolved in DCM (1 mL) in a reaction vail. To this solution of HG2 complex (10 mol%, 8.5 mg) in DCM (0.5 mL) was added. The reaction mixture was stirred at room temperature for 12 h. The polymerization was quenched using ethyl vinyl ether (0.5 mL). Polymer was precipitated into methanol to afford an off-red solid (80 mg, 74%). ^1^H NMR [400 MHz, CDCl_3_] *δ* 7.87 (1 H, s), 7.47 (2 H, s), 7.09 (1 H, s), 6.88 (1 H, s), 6.48–6.31 (4H, m), 6.29 (2 H, d, *J* = 8.4), 6.07 (1 H, s), 5.78 (1 H, s), 5.11 (1 H, s), 4.46 (1 H, s), 3.36 (16 H, d, *J* = 22.8), 2.49 (1 H, s), 2.24 (1 H, s), 1.77 (2 H, s), 1.36 (2 H, s), 1.24 (15 H, d, *J* = 60.1 Hz), 0.88 (1 H, t, *J* = 7.0 Hz); ^13^C NMR (151 MHz, CDCl_3_) *δ* 175.84, 153.46, 153.34, 149.12, 135.04, 130.12, 128.58, 128.25, 124.05, 123.08, 108.49, 108.25, 97.83, 63.10, 47.64, 44.39, 38.84, 27.48, 26.26, 22.21, 21.08, 18.22, 15.41 and 12.62.; IR (ATR) *ν* = 1698, 1676 and 1610 cm^−1^.

## Results and discussion

**Scheme 1:**
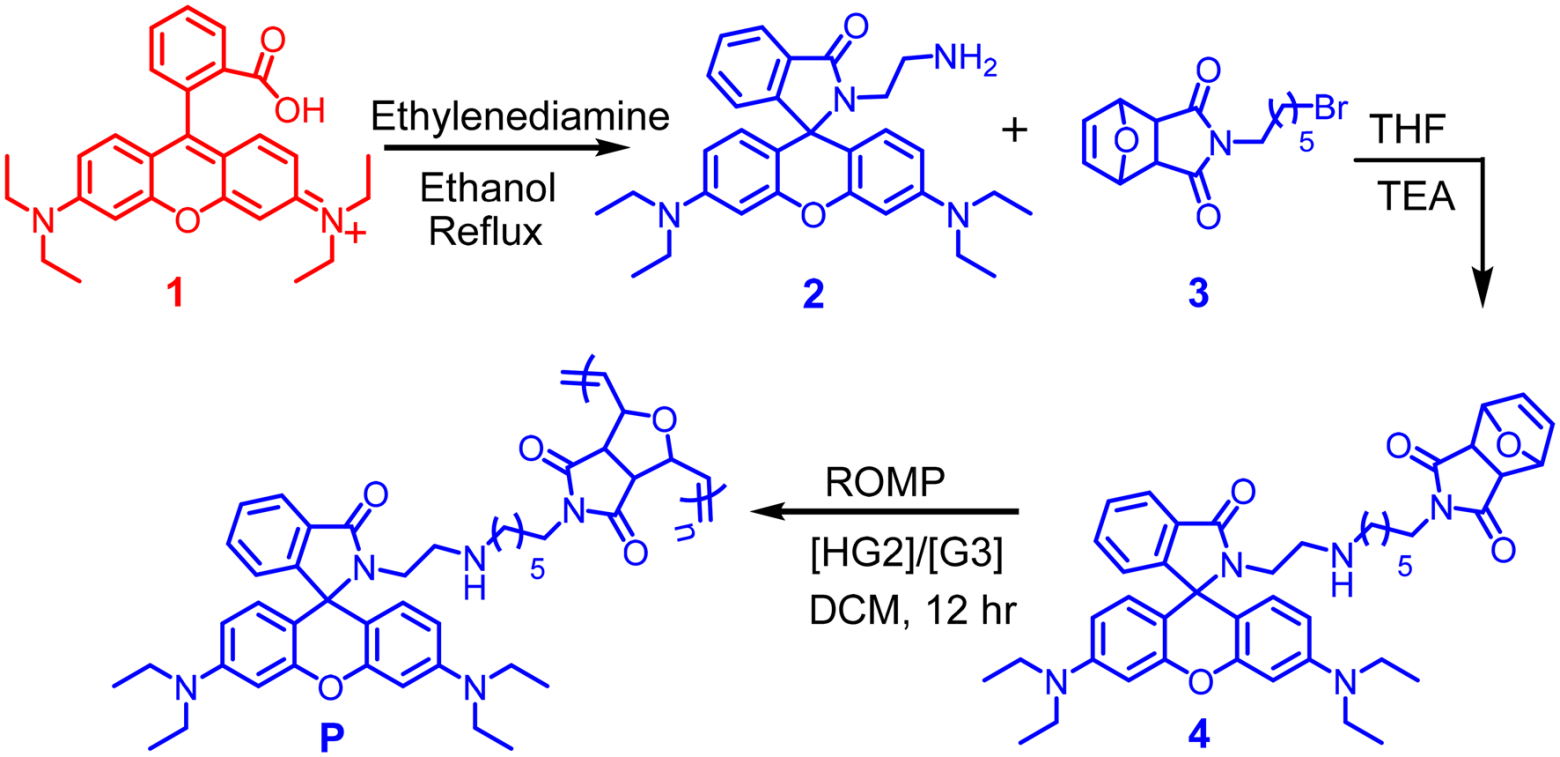
Synthetic route to probe **4** and polymer **P**.

As depicted in Scheme 1 spirolactam intermediate **2** was synthesized by reacting rhodamine B ethylethanaminium **1** with excess ethylene diamine. Subsequent amine alkylation with bromohexyl-dione **3** in THF as the solvent afforded monomer **4** in 54% yield. ROMP of **4** using Hoveyda-Grubbs 2nd generation catalyst (HG2) afforded polymer **P** as light red solid in 74% yield. ^1^H NMR spectroscopy confirmed the full conversion of the monomer **4** to the polymeric material **P**, thus, the olefinic peaks of the monomer **4** at *δ* = 6.50 ppm were replaced by new signals at *δ* = 6.07 and 5.78 ppm correspond to the *cis* and *trans* olefinic double bonds of **P**. Number-average molecular weight (*M*_*n*_) and dispersity (*Đ*) of polymer **P** was measured via gel permeation chromatography (GPC) in THF using polystyrene as a standard. Polymer **P** has a *M*_*n*_ of 913 Da and a polydispersity index of 1.28. Thermogravimetric analysis (TGA) confirmed that **P** has a thermal decomposition temperature (*T*_*d*_) at 10% weight loss of 300 °C, and differential scanning calorimetry (DSC) under an inert atmosphere indicated the glass transition temperature (*T*_*g*_) to be 146 °C, both techniques confirming the stability of **P** (Fig. SI 8).

### Optical properties

**Figure 1:**
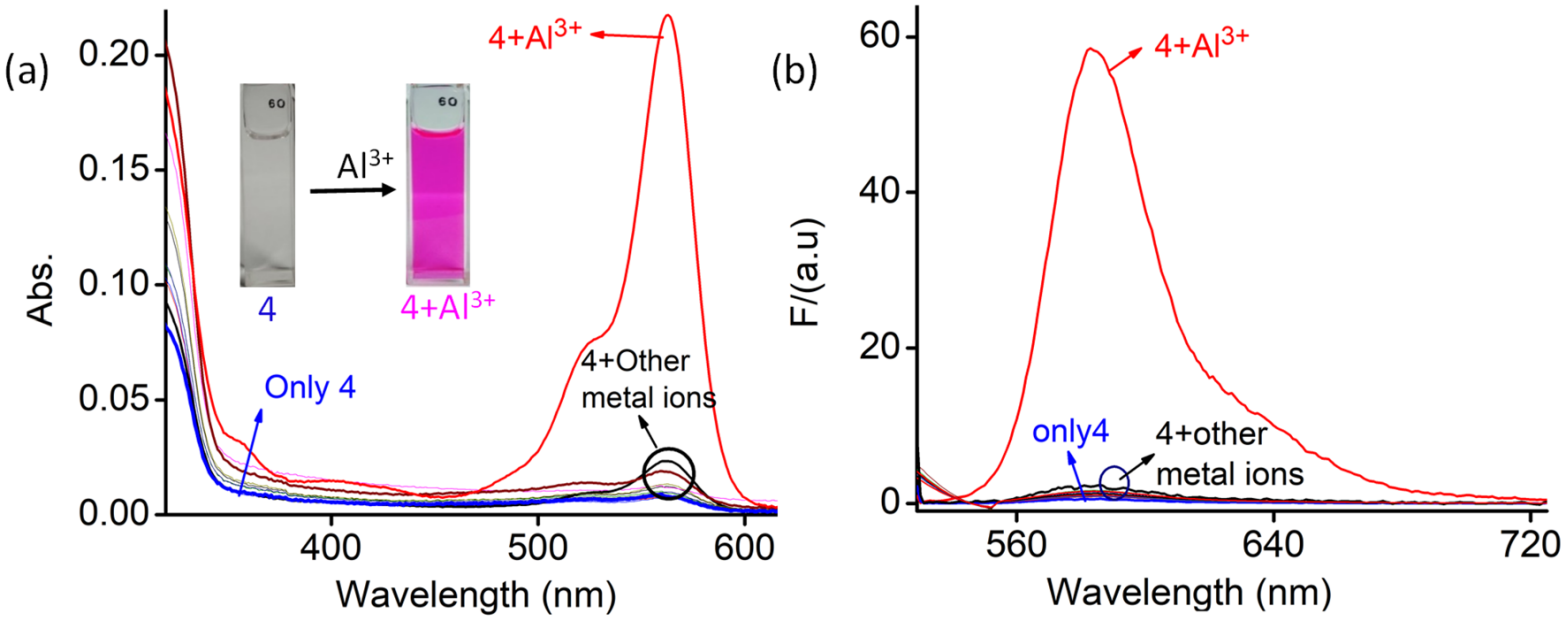
Changes in (**a**) absorption and (**b**) emission spectra (*λ*_Ext_ of 525 nm; slit = 2.5/2.5 nm) of the probe **4** (10 µM) in the absence and the presence of different metal ions (0.9 mM). (M^n+^  = Na^+^, K^+^, Mg^2+^, Ca^2+^, Ba^2+^, Cu^2+^, Ni^2+^, Zn^2+^, Cd^2+^, Co^2+^, Fe^2+^, Cr^3+^, Pb^2+^, Al^3+^ and Hg^2+^) in aq. HEPES buffer-acetonitrile (1:1, v/v; pH 7.2) medium.

Rhodamine derivatives are known to be notorious for binding to certain transition metal ions. Thus, we first turned our attention to examine the absorbance and fluorescence emission properties of probe **4** in the absence and presence of various metal ion, such as Na^+^, K^+^, Mg^2+^, Ca^2+^, Ba^2+^, Cu^2+^, Ni^2+^, Zn^2+^, Cd^2+^, Co^2+^, Fe^2+^, Cr^3+^, Pb^2+^, Al^3+^ and Hg^2+^. UV–Vis and emission spectra of probe **4** and polymer **P** (10 µM) were recorded in aq. HEPES buffer (10 mM)–acetonitrile (1:1, v/v; pH 7.2) medium. The prepared stock solutions of **4** and **P** did not induce any color changes for several weeks, thus suggesting that both materials are stable at room temperature. As depicted in Fig. 1, probe **4** did not show any UV–Vis or emission spectral band beyond 500 nm in absence of any metal ion.

This complete absence of any absorption or emission band in the visible region of the spectrum accounts for the colourless nature in the aqueous solution, hence, strongly confirming that probe **4** is solely in the spirolactam form at neutral condition. This was further confirmed by the ^13^C NMR spectrum, in which a peak appeared at  ~ 67.07 ppm corresponding to the tertiary C-atom of probe **4** (Fig. SI 9). Among the tested metal ions, probe **4** exhibited a large spectral response upon binding with Al^3+^. The UV–Vis spectra showed a distinct change with the appearance of a new absorption band at *λ*_*max*_ = 563 nm with a visually detectable colour change from a colourless solution to a pink solution (Fig. 1a). The appearance of new band in the visible region demonstrates the formation of xanthene framework spawned upon opening of the spirolactam ring due to the complex formation by metal ion chelation. This was confirmed by the ^13^C NMR spectrum, in which the peak at *δ* = 67.07 ppm corresponding to tertiary carbon signal had disappeared upon addition of Al^3+^ to probe **4** in CDCl_3_ solvent (Fig. SI 9). This signified the formation of the acyclic xanthene form. Selective binding of **4** to Al^3+^ among other metal ions was investigated by using fluorescence spectroscopy (Fig. 1b). Among the tested metal ions, a new strong emission band *λ*_max_ at 584 nm appeared only for Al^3+^ upon excitation at *λ*_Ext_ = 525 nm. Thus, the new emission band observed at 584 nm could be ascribed to the binding of Al^3+^ to probe **4**.

**Figure 2:**
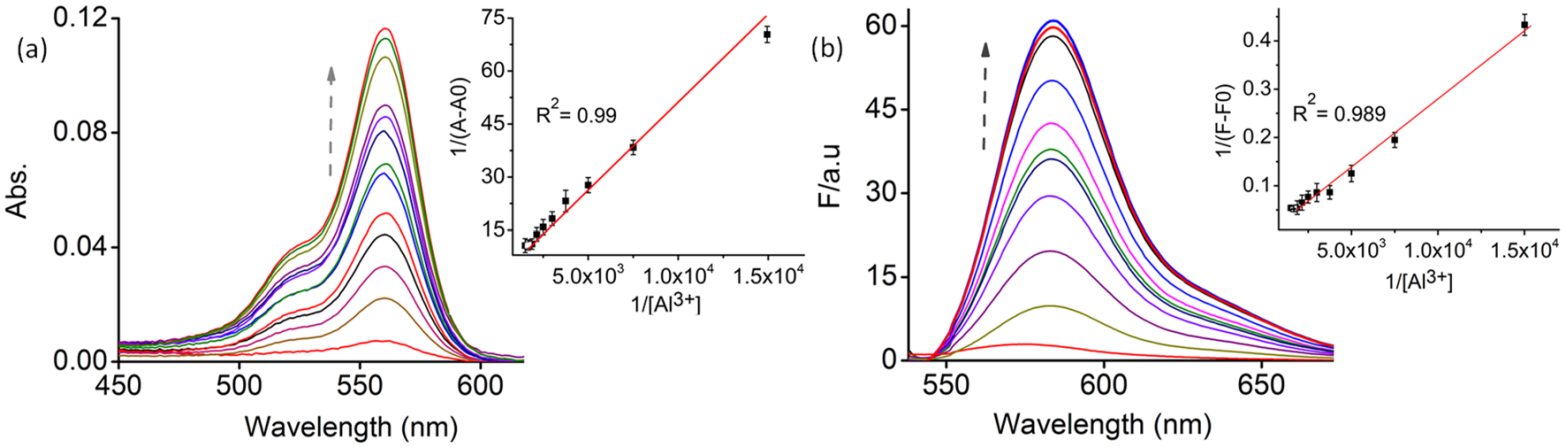
Systematic changes in (**a**) absorption and (**b**) emission (*λ*_Ext_ = 525 nm; slit width 2.5/2.5 nm) spectral patterns for **4** (10 µM) in the presence of varying [Al^3+^] (0–80 µM); Inset Benesi-Hildebrand plot of **4** obtained from UV–Vis and fluorescence titration.

The binding behaviour of Al^3+^ towards **4** was evaluated from the systematic UV–Vis and fluorescence titration experiments (Fig. 2). The Benesi‐Hildebrand (B-H) plot 1/(A – A_0_) and or 1/(F – F_0_) against 1/[Al^3+^] was linear (Fig. 2a,b inset), showing an R^2^ value to be 0.99^54,56^. This linear fit confirms the 1:1 ratio binding stoichiometry between the probe **4** and Al^3+^ and the associated binding constant for the formation of **4**. Al^3+^ concentration was evaluated from the data obtained from B-H plots of the systematic absorption to be *K*_a_^Abs^ = 3.5 × 10^4^ M^−1^ and emission *K*_a_^Ems^ = 3.6 × 10^4^ M^−1^ spectral titrations. In addition, the binding ratio was confirmed using the result from the Job plot obtained from the UV–Vis titration experiments^54^.

**Figure 3:**
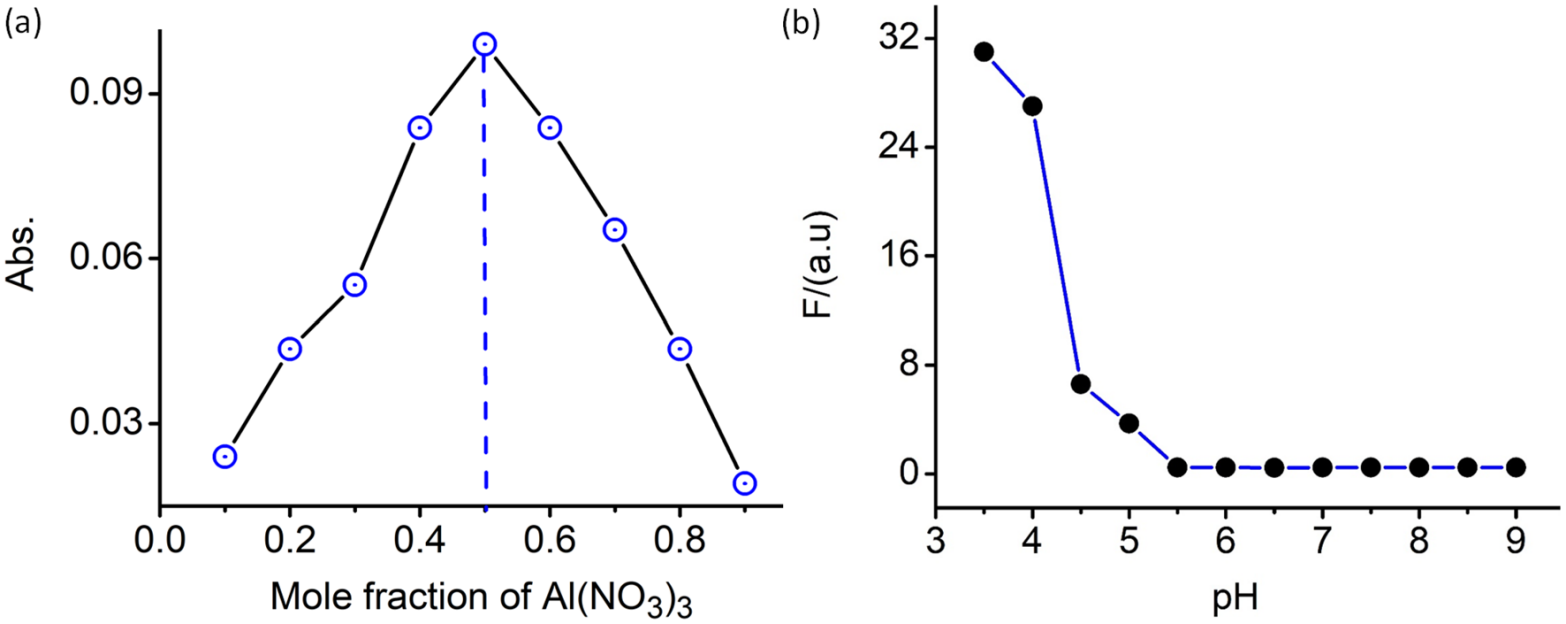
(**a**) Job plot between **4** and Al^3+^ confirmed 1:1 adducts. (**b**) Fluorescence response of **4** (10 µM) as a function of pH in acetonitrile-aqueous buffer (1: 1, v/v).

Figure 3a illustrates the Job plot indicating the maximum mole fraction of Al(NO_3_)_3_ to be 0.5 and reveals the binding of probe **4** to Al^3+^ in 1:1 stoichiometric ratio. The lower limit of detection (LOD) of probe **4** for Al^3+^ [3*σ*/slope] (using data obtained from fluorescence titration) was found to be 2.1 µM, which is lower than the value set by the World Health Organization (WHO) in drinking water of 200 μg L^−1^ (7.41 µM)^7^. To examine the role of probe **4** as a pH sensor, fluorescence spectra for probe **4** was recorded (*λ*_Ext_ = 525 nm) at different pH ranges. The results of the study revealed that the spirocyclic form of probe **4** is relatively stable in the pH range 5.5 to 9.0 (Fig. 3b). This further confirms that the luminescence in response to 584 nm is only due to the specific binding of the probe **4** to Al^3+^. To have a better insight into the possible binding mechanism of probe **4** towards Al^3+^, IR spectra was also recorded in absence and presence of Al^3+^. Stretching frequencies for the C=O (imide) and C=O (amide) groups of probe **4** appeared at ~ 1692 and ~ 1676 cm^−1^. Upon addition of Al^3+^, the band at 1676 cm^−1^ corresponding to C=O (amide) disappeared and another band at ~ 1692 cm^−1^ remained almost invariant upon binding of Al^3+^ to probe **4**. This tend to leave us with an impression that C=O (imide) of the probe **4** was not involved in the coordination with Al^3+^ (Fig. SI 11).

**Figure 4:**
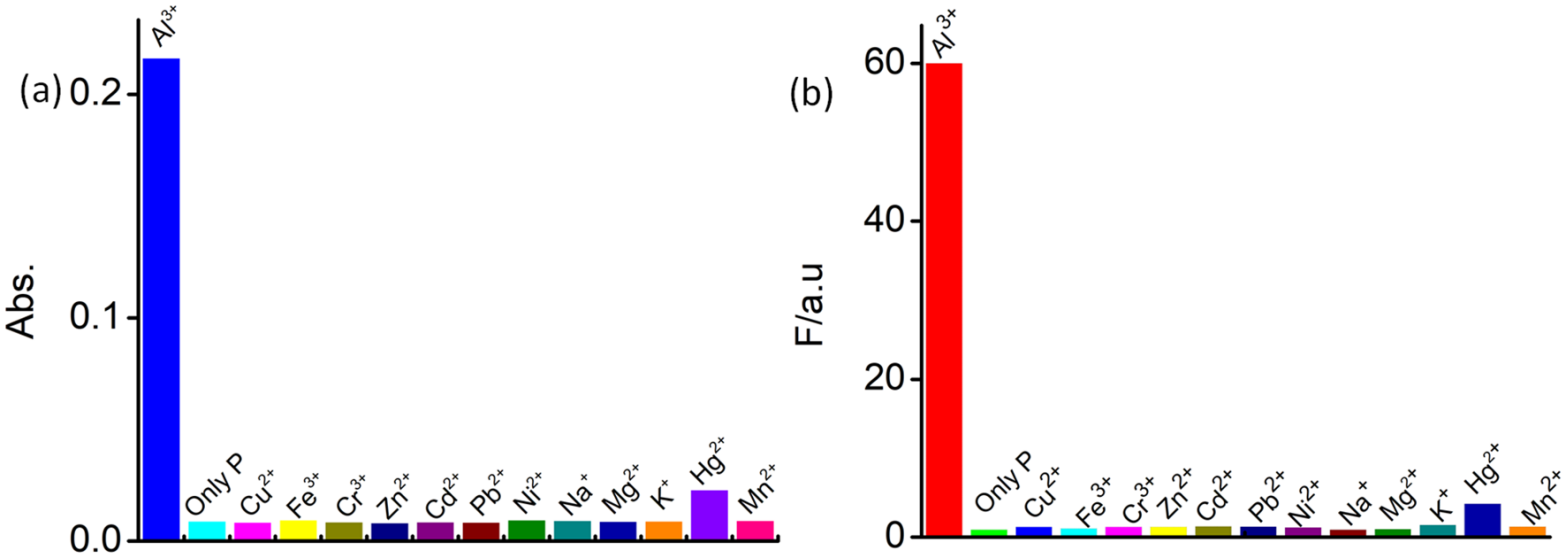
Changes in (**a**) absorption and (**b**) emission spectra (*λ*_Ext_ of 525 nm; slit = 2.5/2.5 nm) of **P** (5 µM) in absence and presence of different metal ions (0.45 mM).

Next, we turned our attention to the sensing behaviour of polymer **P**, which was obtained from probe **4** via ROMP approach. The selective binding of the polymer **P** towards metal ions M^n+^  = Na^+^, K^+^, Mg^2+^, Ca^2+^, Ba^2+^, Cu^2+^, Ni^2+^, Zn^2+^, Cd^2+^, Co^2+^, Fe^2+^, Cr^3+^, Pb^2+^, Al^3+^ and Hg^2+^ was also investigated using UV–Vis and fluorescence studies (Fig. 4a,b). The spectra recorded for polymer **P**, and **P** + M^n+^ (M^n+^  = metal ions except Al^3+^) did not show any change in the absorbance and fluorescence spectra at *λ*_*Ext*_ = 525 nm.

In addition, the Ru^4+^ metal in [HG2] complex did not induce spirolactam ring opening, it only played a catalytic role in the ROMP reaction. To the best of our knowledge, this is first report in the literature that describes rhodamine B appended polymer synthesized via ROMP for sensing of Al^3+^ ions. Similarly to probe **4**, polymer **P** also illustrated sharp changes in the electronic and emission spectra in the presence of Al^3+^ at *λ* = 563 and 584 nm, respectively. In addition, polymer **P** is also invariant to pH in the ranges of 5.5 to 9.0 (Fig. SI 12). Reversible binding of Al^3+^ to polymer **P** was also established with the restoration of the original absorption or emission spectra of polymer **P** by treating solution **P** and Al^3+^ with excess Na_2_EDTA (Fig. SI 13). EDTA^2−^ is a strong chelating agent and known to have a much higher affinity (pKa = 23.6 in aq. medium) toward Al^3+^. Addition of EDTA^-2^ to the resulting **P**-Al^3+^ complex solution led to an immediate decrease in the intensity of absorption and emission band at 563 and 583 nm respectively, indicating the reversible binding nature of the sensor.

Interestingly, LOD of polymer **P** for Al^3+^ detection was found to be 0.5 µM, which is four folds lower than that of probe **4**. This confirms that polymer **P** has a higher sensitivity towards Al^3+^ detection than probe **4**. In addition, intervention studies in the presence of excess metal ions: Al^3+^ (1:1) were carried out to ensure that both probe **4** and polymer **P** have a stronger binding affinity towards Al^3+^ even in the presence of higher concentrations of other competing metal ions (Fig. SI 17). Rhodamine derivatives are also acts as photoactivatable photochromic systems under certain light conditions after being complexed with certain metal ions^57,58^. In this system, we did not observe any color change after addition of metal ions to the solutions of **4** and **P** under day light conditions for few days except for Al^3+^ ion. Suggests that spirolactum ring of **4** and **P** are in closed form.

After confirming in fact that the both **4** and **P** have a stronger binding specificity to Al^3+^ in comparison to the other metal ions, we next evaluated the distribution and behaviour of probe **4** in three different cell lines: human embryonic kidney cells (HEK 293), a breast cancer cell line (MCF-7) and a cervical cancer cell line (HeLa). Cells were loaded with 25 μM of probe **4** and then washed to remove the excess probe.

**Figure 5:**
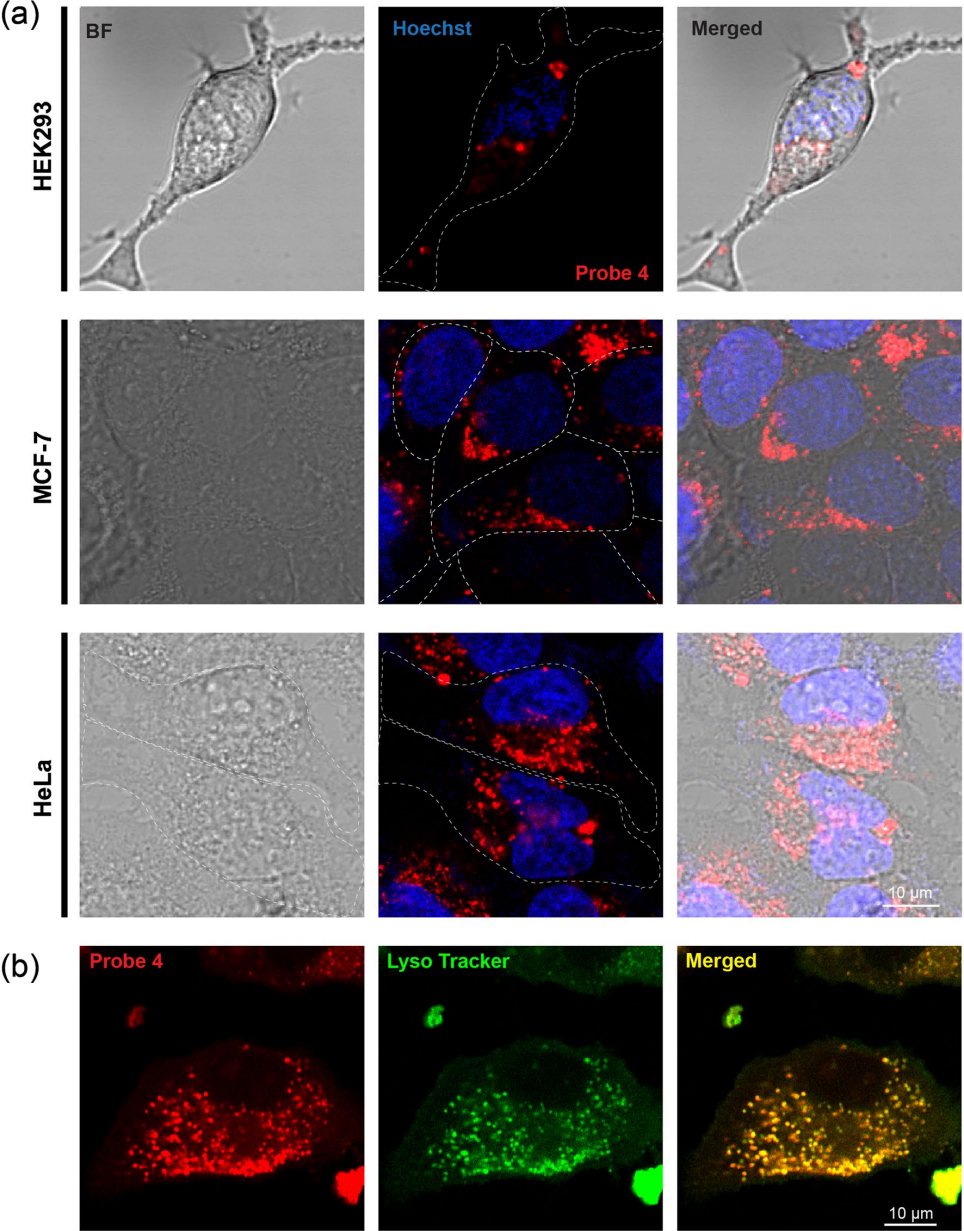
(**a**) Labelling of three cell types (HEK293, MCF-7 and HeLa, indicated on the left) by 25 µΜ of probe **4**. The overall structure of the cell is illustrated on the left using scattered laser light to generate a bright field image (BF), the fluorescence emitted by probe **4** is shown in red and the nuclei stained with Hoechst in blue. (**b**) Co-staining of HeLa cells with probe **4** (5 µΜ, red) and Lysotracker (0.5 µΜ, green). The yellow pattern in the merged image illustrates the co-localization of the two dyes.

Cells were then either left untreated or further treated with Al^3+^ (50 µΜ) to assess whether the probe can detect Al^3+^ in vivo. Intracellular emission of probe **4** was visualized on a scanning confocal platform using the *λ*_Ext_ = 561 nm laser line for excitation. Interestingly, all cell lines showed bright red intracellular emission even in absence of Al^3+^ (Fig. 5a). Furthermore, no changes in emission was observed for **4** after Al^3+^ addition (not illustrated). These observations argue that the emission detected in cells is not due to the binding of Al^3+^, especially since the levels on free intracellular Al^3+^ are not expected to be high and that the affinity of **4** towards Al^3+^ is relatively low. This argues that the basal labelling of intracellular organelles is not Al^3+^ dependent but rather is an inherent property of the probe **4**. The organelle labelling was distinct from that expected for the endoplasmic reticulum or mitochondria and was rather more indicative of lysosomes in all three-cell lines (Fig. 5a). To directly test whether the probe localizes specifically to lysosome, we labelled the cells with the probe **4** and 0.5 μM of Lysotracker Blue DND-22, a specific probe for the lysosome. As illustrated in Fig. 5b the same intracellular structures were labelled by probe **4** and by the lysosomal indicator.

**Figure 6:**
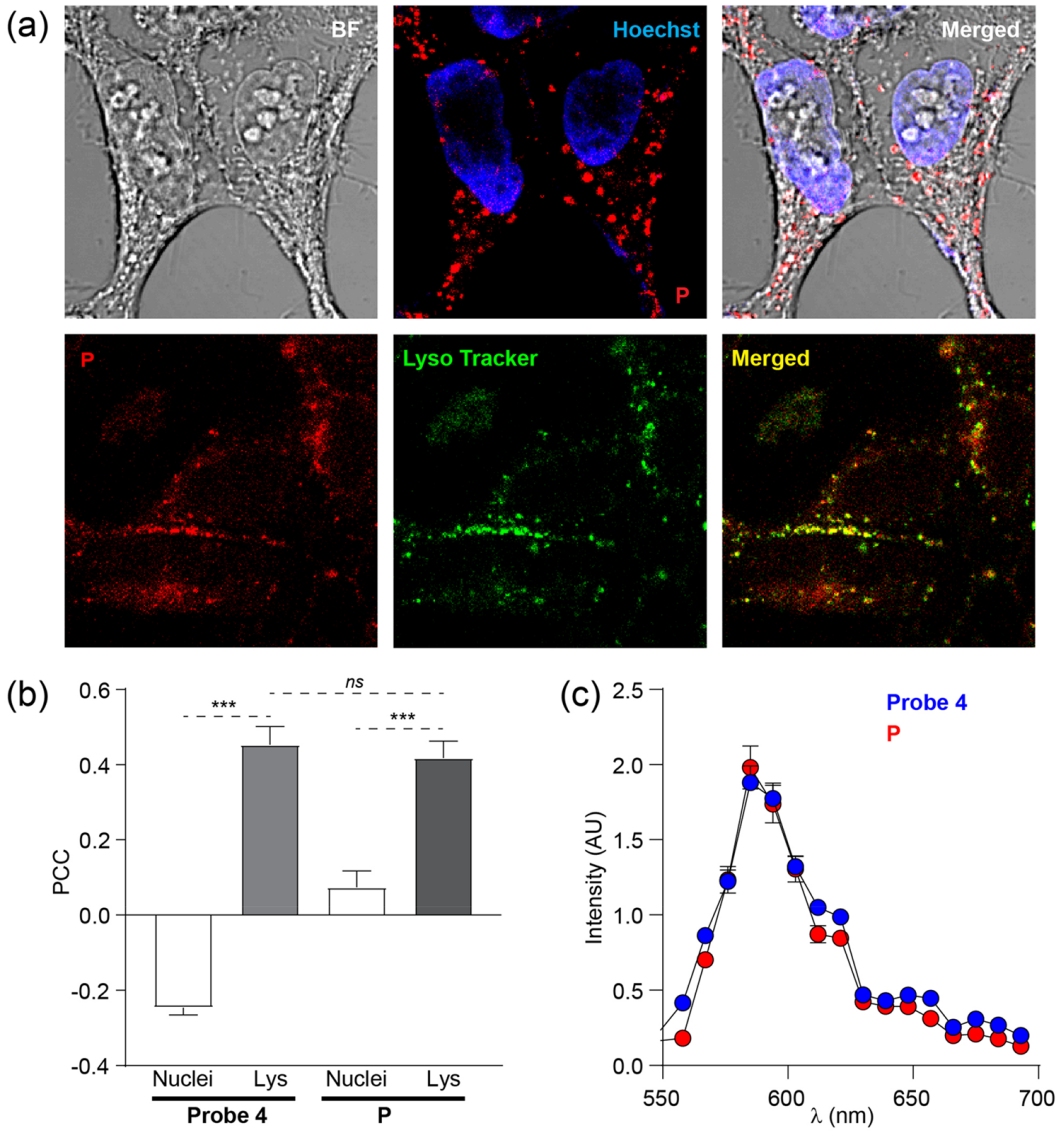
(**a**) The cells were loaded with **P** (5 µΜ, red) and either Hoechst (blue) or lysotracker (green) to reveal the sub-cellular distribution of **P**. (**b**) Quantification of the colocalization of probe **4** and **P** with the nuclei and the lysosome expressed as Pearson’s Correlation Coefficient (PCC). (**c**) Emission spectra of probe **4** and **P** when excited with a 561 nm laser line. Values are mean ± S.E.M, statistics according to Student’s t-test as follows: *ns*: not significant; ***: p < 0.01.

This confirmed that probe **4** specifically labels lysosomes. Following these findings, we next assessed the behaviour of polymer **P** in a cellular environment. As illustrated in Fig. 6a, the staining pattern observed for **P** was very similar to probe **4** showing a dotted/vesicular pattern that colocalizes with the lysosomal marker. We quantified this colocalization by measuring the Pearson’s correlation coefficient (PCC, Fig. 6b) among the probes, specific staining of the nuclei (Hoechst) and of the lysosome (Lysotracker). Both probes show no colocalization (PCC values close to 0 or in the negative range) with the nuclei marker while having significant and comparable colocalization values with the lysosomes (PCC = 0.45 ± 0.04 for probe **4** and 0.42 ± 0.05 for **P**, mean ± S.E.M, n = 10)^59^. By using a lambda scan (sliding emitted wavelength window of 9 nm from 550 to 695 nm), we measured the emission intensity of probe **4** and **P** in vivo under excitation at 561 nm. As indicated in Fig. 6c there was no measurable difference in the optical properties of the two probes. We also measured the decay of fluorescence intensities for both probe **4** and **P** by treating with UV flash (5 s) during cell imaging experiments (Fig. SI 18).

Lysosomes are characterized by a low intraluminal pH about pH 4.5 to 5.0, as compared to the more neutral cytoplasmic pH. Given the known pH sensitivity of **4** we reasoned that probe **4** could be labelling lysosomes specifically because it has inherent pH sensitivity that stimulates its fluorescence. As shown in Fig. 3b, emission intensity of **4** increased dramatically at pH values ≤ 5.0, which is the typical lysosomal pH range (~ 4.5–5.0). These results confirm that probe **4** functions as a lysosomal marker with good cellular permeability. In these cellular labelling studies 5–25 µM of probe **4** were used to label the cells, those concentrations were well tolerated by the cells and displayed no obvious cellular toxicity.

## Conclusions

In summary, oxo-norbornone monomer bearing rhodamine B group **4** was synthesized and characterized. ROMP of **4** afforded polymer **P** in good yields. Both **4** and **P** demonstrated their selectivity for Al^3+^ over other metal ions. The UV–Vis and luminescence properties of **4** and **P** in solution were evaluated in detail. We have also evaluated the distribution and behaviour of probe **4** in three different cell lines: human embryonic kidney cells (HEK 293), a breast cancer cell line (MCF-7) and a cervical cancer cell line (HeLa). In addition, the staining pattern observed for **P** was very similar to probe **4** showing a dotted/vesicular pattern that colocalizes with the lysosomal marker. Intracellular emission of **4** and **P** was visualized on a scanning confocal platform using the λ = 561 nm laser line for excitation. Interestingly, all cell lines showed bright red intracellular emission even in absence of Al^3+^. The UV sensitivity of both molecules suggest that they can be used to track single particles in the cells following photo-activation to reveal the spatiotemporal dynamics of lysosomes.

## Supplementary information

Supplementary Information.
