# Supplementary material for: Multifunctional rhodamine B appended ROMP derived fluorescent probe detects Al3+ and selectively labels lysosomes in live cells
Source: Sci Rep. 2020 Nov 11;10:19519. doi: 10.1038/s41598-020-76525-0 (PMC7658199; doi:10.1038/s41598-020-76525-0)
Supplement: Supplementary file 1 — Supplementary Information. [file 41598_2020_76525_MOESM1_ESM.docx]

**Electronic Supporting Information**

Multifunctional rhodamine B appended ROMP derived fluorescent probe detects Al^3+^ and selectively labels lysosomes in live cells

Upendar Reddy Gandra^1^, Raphael Courjaret^2^, Khaled Machaca^2^, Mohammed Al-Hashimi*^1^ and Hassan S. Bazzi*^1,3^

^1^Department of Chemistry, Texas A&M University at Qatar, P.O.Box 23874 Doha, Qatar;Email:bazzi@tamu.edu; [mohammed.al-hashimi@tamu.edu](mailto:mohammed.al-hashimi@tamu.edu);

^2^Department of Physiology and Biophysics, Weill Cornell Medicine Qatar, P.O. Box 24144, Doha, Qatar

^3^Department of Materials Science & Engineering, Texas A&M University, 209 Reed McDonald Building, College Station, TX 77843-3003, USA

| Table of contents: | Page |
| --- | --- |
| ^1^H , ^13^C NMR and IR spectrum of **2** | S3-S4 |
| ^1^H & ^13^C NMR spectrum of **4** & LCMS spectrum of **4** | S5-S6 |
| ^1^H &^13^C NMR spectrum of **P**; TGA & DSC analysis of **P** | S7-S8 |
| GPC spectrum of **P** | S9 |
| Comparison of partial ¹^3^C NMR spectra of **4** and **P** before and after addition of Al^3+^ | S10 |
| IR spectrum of **4** in absence and presence of Al^3+^; Probable sensing mode of probe **4** with Al^3+.^ | S11 |
| Change in UV of **P** as a function of the solution pH; UV-Vis and fluorescence spectral studies for establishing the reversible binding of Al^3+^ to the **P** | S12 |
| Spectrophotometric interference study of **P** with Al^3+^ in presence of various metal ions& UV and fluorescence response of P in absence and presence of various metal ions | S13 |
| Systematic changes in absorption and emission of **P** and their corresponding Benesi-Hildebrand plots & LCMS spectrum of **4**.Al^3+^ | S14 |
| Modulation of probe **4** and **P** fluorescence in cells by UV light illumination | S15 |
| MTT assay for evolution of cytotoxicity of the probe 4 and P towards Hela cells | S16 |
| Benesi–Hildebrand (B–H) equation for Association constant | S16 |
| **Determination of detection limit** | S17 |

**^1^H NMR spectrum of 2**

Figure S 1. ^1^H NMR spectrum of **2** in CDCl_3_.

**^13^C NMR spectrum of 2**

****Figure S 2. ^13^C NMR spectrum of **2** in CDCl_3_.

**IR spectrum of 2**

Figure S 3. IR spectrum of **2**.

**^1^H NMR spectrum of 4**

Figure S 4. ^1^H NMR spectrum of **4** in CDCl_3._

**^13^C NMR spectrum of 4**

Figure S 5. ^13^C NMR spectrum of **4** in CDCl_3_.

**LCMS spectrum of 4**

Figure S 6. LCMS spectrum of **4**.

**^1^H NMR spectrum of P**

Figure S 7. ^1^H NMR spectrum of **P** in CDCl_3._

**^13^C NMR spectrum of P**

Figure S 8. ^13^C NMR spectrum of **P** in CDCl_3._

**TGA and DSC analysis of P.**

Figure S 9. (a) TGA analysis of **P**. The sample was heated up to 750^o^C under a nitrogen atmosphere at a heating rate of 10 °C/min. (b) DSC analysis of **P** (first heating), sample was heated from 30.00°C to 400.00°C at 10.00°C/min.

**GPC analysis of P**

Figure S 10. GPC analysis of **P** in measured THF solvent.

**Comparison of partial ¹^3^C NMR spectra of 4 before and after addition of Al^3+^**

Figure S 11. Partial ^13^C NMR spectra of **4** in (a) absence and (b) presence of Al^3+^ in CDCl_3_.

**Comparison of partial ¹^3^C NMR spectra of P before and after addition of Al^3+^**

Figure S 12. Partial ^13^C NMR spectra of polymer **P** in (a) absence and (b) presence of Al^3+^ in CDCl_3_.

**IR spectrum of 4 in absence and presence of Al^3+^**

Figure S 13. IR spectra of **4** in absence (black line) and presence (red) of Al^3+^.

**Plausible sensing mode of probe 4 with Al^3+.^**

Figure S 14. Plausible reversible binding mode of probe **4** with Al^3+^.

**Change in UV of P as a function of the solution pH:**

SI Figure 15. UV response of **P** (5 μM) as a function of pH in acetonitrile- buffer (3: 2, v/v), pH is adjusted by using aqueous solutions of 1 M HCl or 1 M NaOH.

**UV-Vis and fluorescence spectral studies for establishing the reversible binding of Al^3+^ to the P**

SI Figure 16. (a) UV and (b) Fluorescence studies for establishing the reversible binding of Al^3+^ (0.45 mM) to **P** (5 μM) in presence of excess EDTA^2-^ using λ_Ext_ = 525 nm; and slit width 2.5/2.5 nm.

**Spectrophotometric interference study of P with Al^3+^ in presence of various metal ions**

SI Figure 17. Spectrophotometric interference study of **P** (5 μM) with Al^3+^ (0.45 mM) in presence of various metal ions (0.45 mM) in acetonitrile by using λ_Ext_ = 525 and λ_EM_^Mon^ = 584nm.

**UV and fluorescence response of P in absenene and presence of various metal ions**

SI Figure 18. Changes in (a) absorption and (b) emission spectra (*λ*_Ext_ of 525 nm; slit = 2.5/2.5 nm) of **P** (5 μM) in absence and presence of different metal ions (0.45 mM).

**Systematic changes in absorption and emission of P and their corresponding Benesi-Hildebrand plots**

SI Figure 19. Systematic changes in (a) absorption and (b) emission (*λ*_Ext_ = 525 nm; slit width 2.5/2.5 nm) spectral patterns for **P** (5 μM) in the presence of varying [Al^3+^] (0–65 μM); Inset Benesi-Hildebrand plot of **P** obtained from UV-Vis and fluorescence titration.

**LCMS spectrum of 4.Al^3+^**


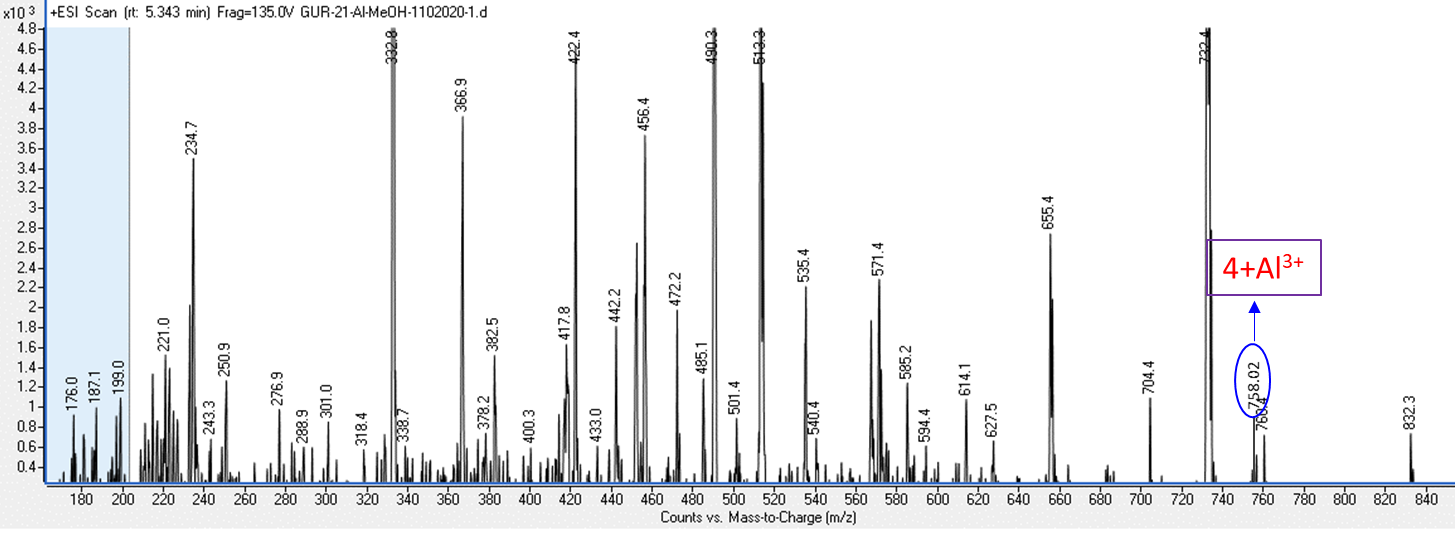


SI Figure 20. LCMS spectrum of **4**.Al^3+^.

**Modulation of probe 4 and P fluorescence in cells by UV light illumination**

**
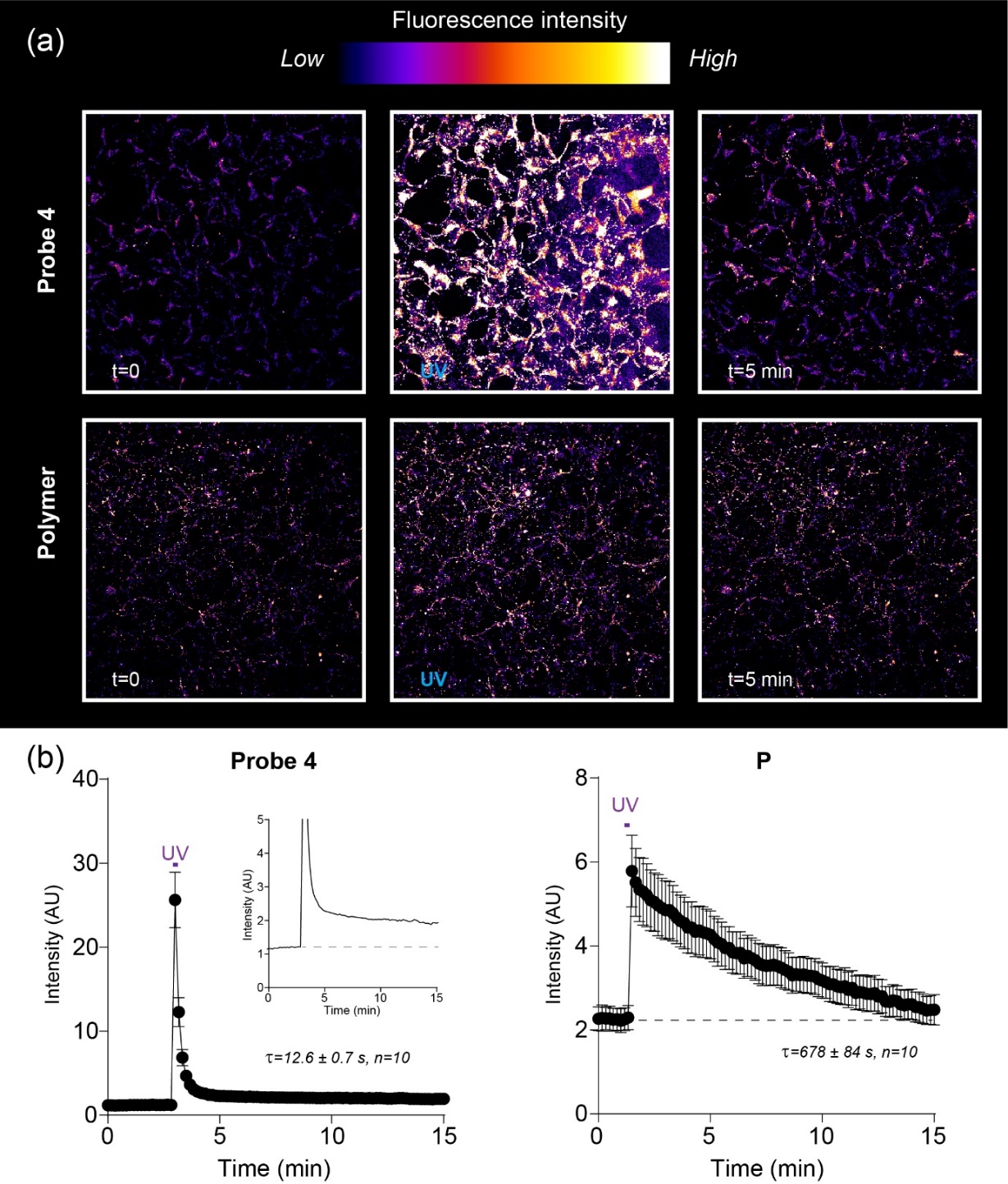
**

SI Figure 21. UV activation of probe **4** and **P** (a) HEK293 cells were labelled with 5 μM of probe **4** and **P** for an hour and imaged at a frequency of 0.1 Hz using the 561 nm laser line of the confocal microscope. UV illumination was produced for 5s using a HBO100 mercury arc lamp connected to the epifluorescence port of the microscope. b) Intensities curves were generated from 10 different cells in the field of view to illustrate the evolution of the fluorescence over time. Upon 5s of UV flash both **4** and **P** showed fluorescence emission enhancement (~25 folds for probe 4 and ~2 folds for **P**). The decay of the fluorescence was different between the two probes: Probe **4** has a much faster decay than **P**, and P fluorescence was returning to the baseline after 10 minutes while probe **4** reached a steady plateau above resting values.

**MTT assay for evolution of cytotoxicity of the probe 4 and P towards Hela cells**


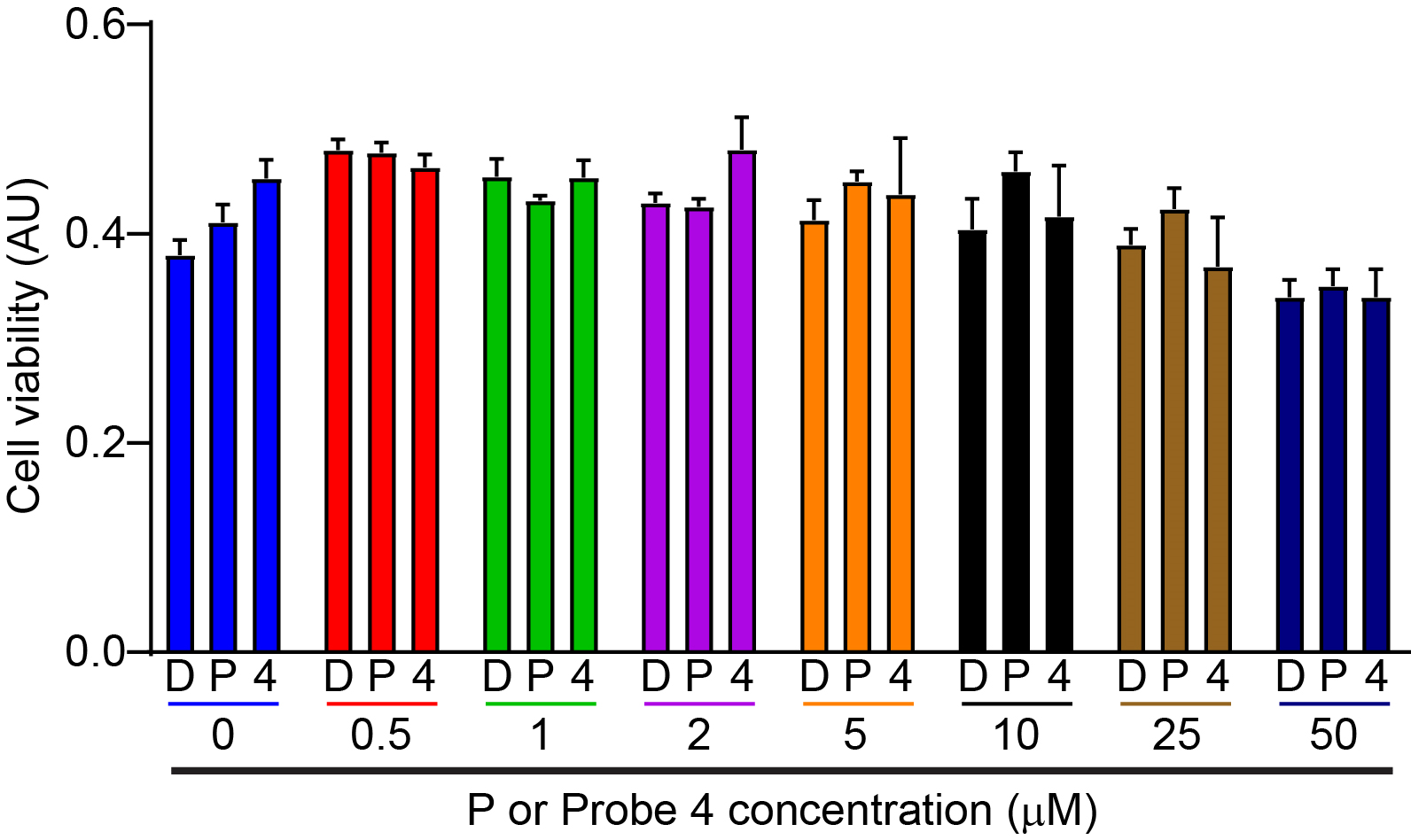


SI Figure 22. Cell viability Assay: Changes in the cell viability measured using an MTT assay after a 1 hour incubation with probe **P** or **4** (in μM) or the corresponding vehicle concentration (DMSO, D). The data have been collected from a single experiment and 4 technical replicates and represented as means ± S.E.M. MTT experiments did not indicate a cytotoxic effect of the probes.

**Benesi–Hildebrand (B–H) equation for Association constant:**

The association constant for the formation of the complex of **4** and **P** with Al^3+^ was determined using the Benesi–Hildebrand (B–H) equation.

where A_0_ is the absorbance maximum of probe **4** or **P**, A is the observed absorbance at that particular wavelength at different concentrations of metal ion [Al^3+^] , A_max_ is the maximum absorbance value at *λ*_max_ = 563 nm (for Al^3+^) during titration with varying [C], *K* is the association constant and was determined from the ratio of slope and intercept of the linear plot, and [C] is the concentration of the Al^3+^ ion added during the titration studies. The goodness of the linear fit of the B–H plot of *1/(A - A_0_)* vs. 1/[Al^3+^] for 1 : 1 complex formation confirms the binding stoichiometry between the probe **4** or **P** with Al^3+^.

The binding constant for the the formation of the complex of **4** and **P** with Al^3+^ was also determined using the Benesi–Hildebrand relation from spectrofluorometric titration.

where [M^n+^] is the metal ion concentration, *F_0_, F* and *F_1_* indicate the fluorescence emission intensities in the absence of, and at intermediate and infinite concentrations of the metal ions, respectively. For 1 : 1 complexation, *m* = 1 and for 1 : 2 complexation, *m* = 2.

**Determination of detection limit**:

The detection limit was calculated based on the fluorescence titration. To determine the S/N ratio, the emission intensity of probe **4** and **P** without Al^3+^ was measured by 10 times and the standard deviation of blank measurements was determined. The detection limit (DL) of probe **4** and **P** for

Al^3+^ was determined from the following equation:

DL = K * Sb1/S

Where K = 2 or 3 (we take 2 in this case); Sb1 is the standard deviation of the blank solution;

S is the slope of the calibration curve.

SI Figure 23. (a) Fluorescence intensity of probe **4** (10 μM) upon addition of Al^3+^ (0-10 μM); (b) Fluorescence intensity of **P** (5 μM) upon addition of Al^3+^ (0-5 μM).
